# MAGI2‐AS3 rs7783388 polymorphism contributes to colorectal cancer risk through altering the binding affinity of the transcription factor GR to the MAGI2‐AS3 promoter

**DOI:** 10.1002/jcla.23431

**Published:** 2020-06-12

**Authors:** Xi Yang, Shenshen Wu, Xiaobo Li, Ying Yin, Rui Chen

**Affiliations:** ^1^ Key Laboratory of Environmental Medicine Engineering Ministry of Education School of Public Health Southeast University Nanjing China; ^2^ Department of Gastroenterology Zhongda Hospital School of Medicine Southeast University Nanjing China

**Keywords:** colorectal cancer, lncRNA, MAGI2‐AS3, polymorphisms, rs7783388

## Abstract

**Background:**

It has been indicated that the single nuclear polymorphisms (SNPs) in the long noncoding RNA (lncRNA) have association with colorectal cancer (CRC) susceptibility.

**Methods:**

We enrolled 1078 cases with CRC and 1175 age‐ and gender‐matched cancer‐free controls to explore whether the polymorphisms in MAGI2‐AS3 have associations with CRC risk. qRT‐PCR, expression quantitative trait loci (eQTL) analyses, dual‐luciferase reporter assay, chromatin immunoprecipitation (ChIP), flow cytometry, and transwell assays were performed to explore the specific mechanisms in which MAGI2‐AS3 rs7783388 variation influenced the tumorigenesis of CRC.

**Results:**

Subjects carrying rs7783388 GG genotype presented a higher risk of CRC compared with the AG/AA genotypes. Mechanistically, we found that the functional genetic variant of rs7783388 A > G decreased binding affinity of transcription factor glucocorticoid receptor (GR) to the MAGI2‐AS3 promoter, resulting in decreased transcriptional activity that subsequently downregulated MAGI2‐AS3 expression. Furthermore, functional experiments elucidated that MAGI2‐AS3 overexpression suppressed CRC cell proliferation, migration, and invasion capacities, arrested cell cycle at G0/G1 phase, and promoted cell apoptosis.

**Conclusion:**

Taken together, our study demonstrated that the potential function of MAGI2‐AS3 as a tumor suppressor for CRC, and the MAGI2‐AS3 rs7783388 polymorphism is associated with the increased susceptibility to CRC by altering the binding ability of GR to the MAGI2‐AS3 promoter.

## 1 INTRODUCTION

Colorectal cancer (CRC) has become a common malignancy that is well known for its poor survival outcomes and advanced metastasis.^1^, ^2^, ^3^ Apart from environmental factors such as obesity, diet, and physical inactivity,^4^, ^5^, ^6^ genetic and epigenetic alternations have been well‐recognized as risk factors in CRC etiology.^7^, ^8^, ^9^, ^10^

Long noncoding RNAs (lncRNAs) are a class of RNA transcripts with a length of over 200 nucleotides and without protein‐coding capacity.^11^ Accumulated evidences have reported that the aberrant expression of lncRNAs exerts their crucial effects on growth, proliferation, metastasis, and angiogenesis of various cancer processes.^12^, ^13^ lncRNA MALAT1 induces colon cancer development by regulating miR‐129‐5p/HMGB1 axis.^14^ Reduced expression of lncRNA H19 inhibits pancreatic cancer metastasis.^15^ It has been proved that the functional single nucleotide polymorphisms (SNPs) within lncRNAs are capable of affecting the susceptibility of cancers.^16^, ^17^, ^18^ Some lncRNA polymorphisms have been used to predict risk of CRC. For example, lncRNA PCAT1 rs2632159,^19^ lncRNA GAS5 rs55829688,^20^ and lncRNA H19 rs2839698 ^21^ have been associated with the increased risk of CRC. lncRNA HOTAIR rs7958904,^22^ lncRNA H19 rs2839698,^23^ and lncRNA RP11‐3N2.1 rs13230517^24^ have been linked to the reduced risk of CRC. MAGI2‐AS3 is a newly discovered lncRNA, and its biological functions remain elusive. Several studies have reported the potential of MAGI2‐AS3 as a tumor suppressor in various cancers.^25^, ^26^, ^27^ However, a recent report indicated a controversial role of MAGI2‐AS3 played in the progression of colorectal cancer.^27^ Therefore, further study on the role of MAGI2‐AS3 in CRC is necessary and we hypothesized that the functional polymorphisms in MAGI2‐AS3 might be contributable to CRC susceptibility.

In this study, a hospital‐based case control was designed to investigate whether the selected polymorphisms in MAGI2‐AS3 influence the susceptibility of CRC, and further uncover the underlying mechanisms.

## 2 MATERIALS AND METHODS

### 2.1 Participants

The case‐control study was designed as previous studies reported.^29^, ^30^ We recruited 1078 consecutive patients with newly diagnosed and histologically confirmed CRC from the Hospital of Jiangsu Province from January 2007 to October 2011. 1175 participants who attended the physical examination in the same hospital were randomly selected as controls. After the physical examination, the controls were determined to be no colorectal cancer and had no biological association with the case group. The detailed information on the study subjects was concluded in Table S1 which has been reported in our previous study.^29^ The frequency distributions of age and gender in controls were matched with cases, and the difference was not statistically significant.

Additionally, 200 patients with CRC were enrolled in our research. CRC tissues and the corresponding adjacent tissues were abstained from patients by surgery in the Affiliated Hospital of Xuzhou Medical University and the Jiangsu Cancer Hospital between 2014 and 2015 to measure the expression of MAGI2‐AS3 levels in CRC. Every patient signed an informed consent and participated in the study voluntarily.

### 2.2 Selection of candidate SNPs

We retrieved the location of MAGI2‐AS3 containing 2 kb of both upstream and downstream flanking sequences using the online database UCSC (http://genome.ucsc.edu/). The candidate SNPs in lncRNA MAGI2‐AS3 were selected using Haploview 4.2 software. The criteria are as follows: (a) the minor allele frequency (MAF) of selected SNPs more than 0.05 in the Chinese population; (b) the *P*‐value for the Hardy‐Weinberg equilibrium (HWE) >0.05; and (c) the level of linkage disequilibrium (LD) of *r* ^2^ < 0.8. Subsequently, two SNPs were selected as candidate targets, including rs7783388 and rs4730857.

### 2.3 DNA extraction and genotype analysis

The Relax Gene Blood DNA System (Tiangen Biotech,) and EZNA Tissue DNA Kit (Omega Bio‐Tek) were used to extract and purify genomic DNA from peripheral blood samples of healthy controls and from paraffin‐embedded sections of CRC patients according to the manufacture's introduction. The genotyping of the candidate SNPs was performed by the TaqMan SNP Genotyping assays equipped with Quant Studio 6 Flex System (Applied Biosystems). Sequencing was performed for 10% randomly selected samples for genotyping confirmation.

### 2.4 RNA solution and qRT‐PCR

Total RNA samples from CRC cells or frozen CRC tissues were extracted using TRIzol reagent (Invitrogen). After that, 1 µg RNA/sample was reversed‐transcribed into first‐strand cDNA with PrimeScript™ RT Reagent Kit (Takara). SYBR Green Real‐time PCR Master Mix‐Plus kits (Toyobo) was used to perform qPCR in triplicate to test MAGI2‐AS3 and GR mRNA level. 2^‐ΔΔCt^ method was used for relative expression calculation. The experiment was carried out in triplicates. Primers used were listed here:

MAGI2‐AS3: 5′‐ATACAAGCCCAAGTTCTG‐3′(sense);

MAGI2‐AS3: 5′‐TTCCTGGTGTTTCCTCTT‐3′ (antisense);

GR: 5′‐TGCGTCTTCACCCTCACT‐3′ (sense);

GR: 5′‐CCAGGTCATTTCCCATCA‐3′ (antisense);

β‐actin: 5′‐ATCCGCAAAGACCTGT‐3′ (sense);

β‐actin: 5′‐GGGTGTAACGCAACTAAG‐3′ (antisense);

### 2.5 Cell culture

We obtained the RKO, SW480, and SW620 cells from Shanghai Institute of Biochemistry and Cell Biology, Chinese Academy of Sciences (Shanghai, China). All cells were maintained at 37°C in advanced Dulbecco's modified Eagle medium (DMEM, HyClone) in a humidified atmosphere containing 5% CO_2_. All medium was supplemented with 10% fetal bovine serum (FBS) (Sigma) and antibiotics (penicillin [HyClone]).

### 2.6 Cell transfection

The sequence of full length of MAGI2‐AS3 was subcloned into PGMLV‐CMV‐MCS‐PGK‐Puro vector (Genomeditech) to construct MAGI2‐AS3 overexpression plasmid. The sequence of full length of GR was cloned into pCDNA3.1 (+) vector (Gene Create) to construct GR overexpression plasmid. Lipofectamine 2000 (Invitrogen) was used to transfect MAGI2‐AS3, GR overexpression plasmid, or the corresponding negative controls into CRC cells, following the manufacture's protocol.

### 2.7 Dual‐Luciferase reporter assays

Reporter plasmids containing MAGI2‐AS3 rs77843388 A or rs7783388 G allele were constructed from Gene Create Biological Engineering Co., Ltd. A total of 3 × 10^5^ SW480 or SW620 cells were planed into 6‐well cell culture plates. Reporter plasmids were co‐transfected with GR overexpression plasmid (or GR negative control plasmid) (Gene Create) and pRL‐SV40 (Promega) by Lipofectamine 2000 Reagent (Invitrogen). The relative luciferase activities were analyzed using the Dual‐Luciferase Reporter Assay Kit (Promega) via FLUO star Omega (Berthold).

### 2.8 Chromatin immunoprecipitation assays

5 × 10^7^ white blood cells in human peripheral were cross‐linked at room temperature with 4% paraformaldehyde for 10 minutes. After incubation, 125 mmol/L fresh glycine was added to stop cross‐linking. ChIP‐ITTM Express Magnetic Assay Kit (Cat. No. 53009, Active Motif) was used to perform ChIP. Chromatin fragments were generated by sonicating the cell lysates and then immunoprecipitated with the GR antibody (Ab2768, Abcam) or control IgG antibody (Cat. No. NI01, EMD Chemicals, Inc). Real‐time PCR amplification was carried out using 2 µL of DNA sample with primers (human MAGI2‐AS3 promoter bearing GR binding site), F: 5′‐ACCCAAGTGGTTCGGCTCT‐3′ and R: 5′‐TCCTGCTCCGTTTGTTTA‐3′ specific to the MAGI2‐AS3 promoter.

### 2.9 Cell proliferation analysis

A total of 5 × 10^3^ SW480 and SW620 cells transfected with MAGI2‐AS3 overexpression or negative control plasmid were inoculated into 96‐well plate. After the cell inoculation for 1, 2, and 3 days, CCK‐8 (Beyotime Biotechnology) reagent (10 µL) was added into cells and sustained for 1 hour away from light at 37°C. Cell viability was reflected via detecting the absorbance at 450 nm at each time point.

### 2.10 Cell migration and invasion test

For invasion assays, 5 × 10^4^ transfected cells were planed into the top transwell chamber (Corning) coated with Matrigel (1:20, BD Corning) for around 2 hours before cell inoculation. Cells in the top chambers were suspended in 250 µL medium without serum. The lower chambers were filled with 550 µL medium complemented with serum. After 24 hours, cells invade to the bottom surface were fixed with 4% paraformaldehyde and stained by crystal violet for 15 minutes. Cell migration assay was performed in a similar fashion, but without Matrigel in the transwell chambers.

### 2.11 Flow cytometry

The apoptotic rate of CRC cell was detected using Annexin V‐FITC Apoptotic Detection Kit (Invitrogen). After cell transfection for 48 hours, cells were resuspending in 500 µL ice‐cold binding buffer and then incubated with 5 µL Annexin V‐FITC for 30 minutes, and then stained with 10 µL PI away from light at room temperature for 5 minutes. As for cell‐cycle analysis, after cells washed with cold PBS at 4°C for twice, 70% ethanol was added into each sample to fix cells at −20°C overnight. The cells were incubated with 10 µL RNase for 30 minutes at 37°C. Finally, the cells were incubated with 50 µL propidium iodide (PI) solution for 15 minutes away from light. Cell ‐cycle and apoptosis analysis were detected through the fluorescence‐activated cell sorting (FACS) flow cytometer (BD Biosciences).

### 2.12 Western blot

Proteins extracted from cells were separated by 10% SDS‐PAGE (Beyotime) and then blotted onto PVDF membranes (Millipore) using a wet transfer method. The membrane was incubated overnight at 4°C with the primary antibody: anti‐GR antibody (Ab2768, Abcam), anti‐cleaved caspase 3 antibody, anti‐cleaved PARP antibody (Cell Signaling Technology), and β‐actin antibody (CMCTAG). Membranes were exposed to HRP‐conjugated secondary antibody (Cell Signaling Technology) for 1 hour at room temperature and reacted by using EZECL Chemiluminescence Detection Kit (Millipore) for 1 minute at room temperature.

### 2.13 Statistical analysis

SAS 9.4 (SAS Institute) was applied to perform all the statistical analyses. The independent segregation of the alleles was confirmed by the Hardy‐Weinberg equilibrium (HWE) test. Chi‐square test was performed to analyze the difference among cases and controls. The association between lncRNA MAGI2‐AS3 rs7783388 and rs4730857 and CRC susceptibility was assessed by multivariate logistic regression adjusted for age and gender. Two‐tailed Student's *t* test was conducted to compare groups' pairs. Difference was considered significantly when *P*‐value <.05.

## 3 RESULTS

### 3.1 Association between the selected polymorphisms in MAGI2‐AS3 and CRC susceptibility

Two genetic variants (rs7783388 and rs4730857) located in MAGI2‐AS3 were genotyped. The distribution of alleles of the rs7783388 and rs4730857 polymorphisms among controls adheres to the expectations of HWE (*P* = .9852 for rs7783388 and 0.2152 for rs4730957) (Table 1). The results showed that compared to the rs7783388 AA genotype, the rs7783388 GG genotype had association with a 76% increased risk of CRC (GG vs AA, *P* < .001, adjusted OR = 1.76, 95% CI = 1.38‐2.25) (Table 1). Moreover, our results indicated that the genotype frequency distributions of SNP rs7783388 in a genotype recessive model showed increased risk of CRC (GG vs AG/AA, *P* < .001, adjusted OR = 1.90, 95% CI = 1.51‐2.38). However, it suggested no significant association between genotype distribution of the rs4730857 polymorphism and CRC risk in our population.

**TABLE 1:** Associations between lncRNA polymorphisms and colorectal cancer risk

Genotype		Cases^a^		Controls^a^		*P*	Adjusted OR (95% CI)^b^
		n	%	n	%		
rs7783388	AA	426	40.1	482	41.5	**<.0001**	1.00 (ref)
	AG	405	38.2	533	45.9		**0.86 (0.72‐1.03)**
	GG	230	21.7	147	12.6		**1.76 (1.38‐2.25)**
	AA/AG	831	78.3	1015	87.4	**<.0001**	1.00 (ref)
	GG	230	21.7	147	12.6		**1.90 (1.51‐2.38)**
	G allele		0.4076		0.3558	.0082	
	HWE				0.9852		
	P trend					.0007	
rs4730857	CC	565	53.0	571	52.8	.9556	1
	CT	415	38.9	418	38.7		1.00 (0.84‐1.20)
	TT	87	8.1	92	8.5		0.96 (0.70‐1.32)
	T allele		0.2760		0.2784	.1359	
	HWE				0.2152		
	P trend					.8605	

Note

*P*‐value <.05 was considered statistically significant (in bold).

Pearson's chi‐square test for difference in distributions between the case and control groups.

^a^ The mismatch between the number of genotyping samples and a total of samples is due to the absence of samples.

^b^ Adjusted for age and gender in the logistic regression model.

### 3.2 rs7783388 A > G polymorphism impairs MAGI2‐AS3 expression

**FIGURE 1:**
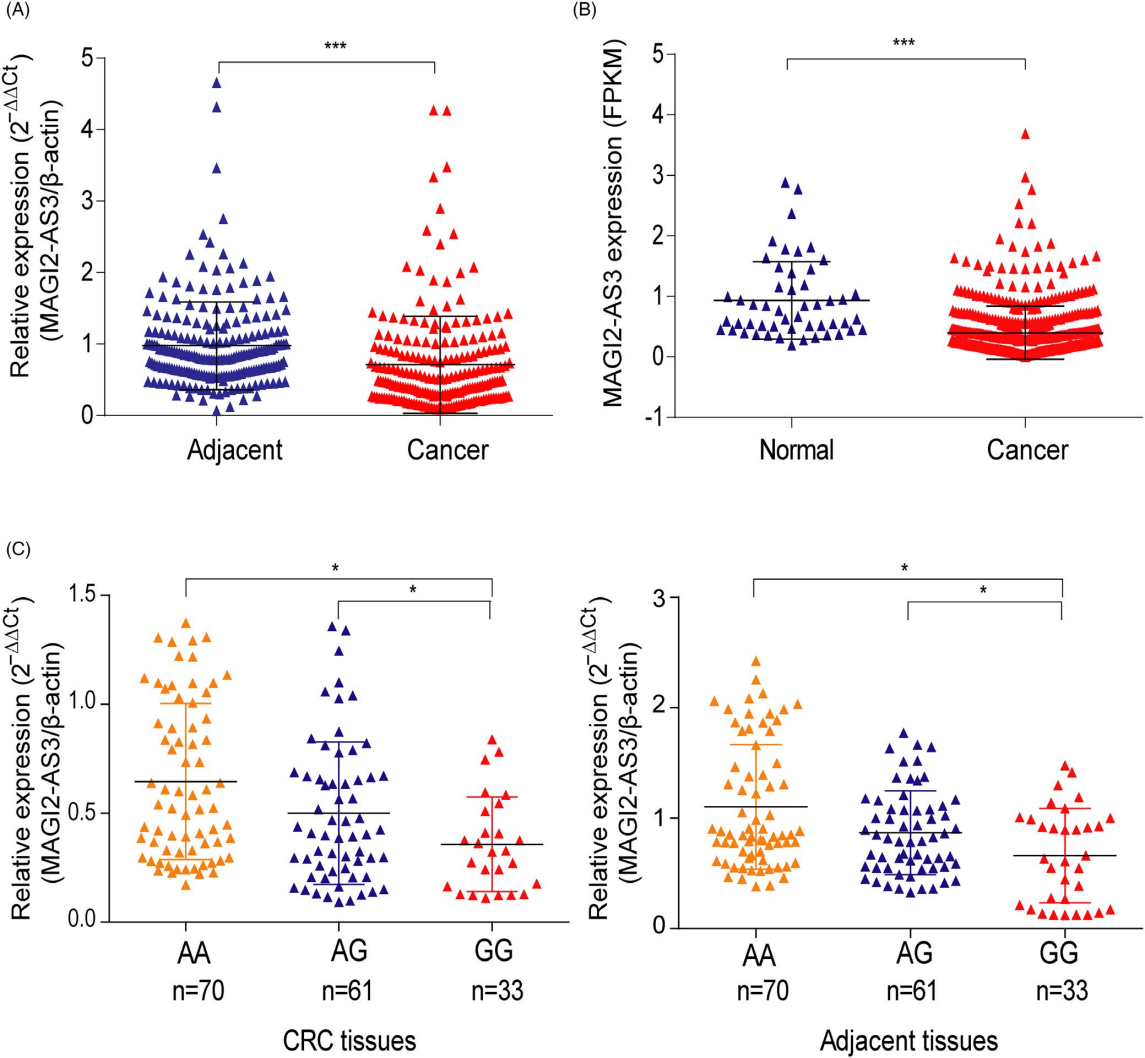
MAGI2‐AS3 is down‐expressed in human CRC. A, Relative expression of MAGI2‐AS3 in 200 paired CRC tissues and adjacent tissues was determined by RT‐qPCR. B, Expression of MAGI2‐AS3 647 CRC tissues vs 51 normal tissues derived from TCGA database. C,MAGI2‐AS3 expression was determined by RT‐qPCR in CRC tissues and adjacent tissues in patients with AA (n = 70), AG (n = 61), or GG (n = 33) genotype at rs7783388. *P < .05, ***P < .001

We first detected the expression of MAGI2‐AS3 in CRC by qRT‐PCR and found that MAGI2‐AS3 expression was significantly decreased in CRC tissues compared with adjacent tissues (Figure 1A). Lower level of MAGI2‐AS3 was also observed in CRC tissues than normal tissues by retrieving TCGA database (Figure 1B). Furthermore, patients with rs7783388 AG/AA genotypes presented higher MAGI2‐AS3 levels than those with the rs7783388 GG genotype in CRC tissues, as well as in adjacent tissues (Figure 1C). We also found a higher MAGI2‐AS3 level in esophageal muscularis tissues in subjects carrying with rs7783388 AG/AA genotypes than those with GG genotype by eQTL analysis in Genotype‐Tissue Expression (GTEx) project (Figure S1A). Moreover, rs7783388 AG/AA genotypes increased the level of MAGI2‐AS3 in colon tissues (Figure S1B). Taken together, rs7783388 A > G variant decreased expression level of MAGI2‐AS3 in CRC and adjacent tissues.

### 3.3 rs7783388 GG genotype decreased the binding affinity of GR to the MAGI2‐AS3 promoter

**FIGURE 2:**
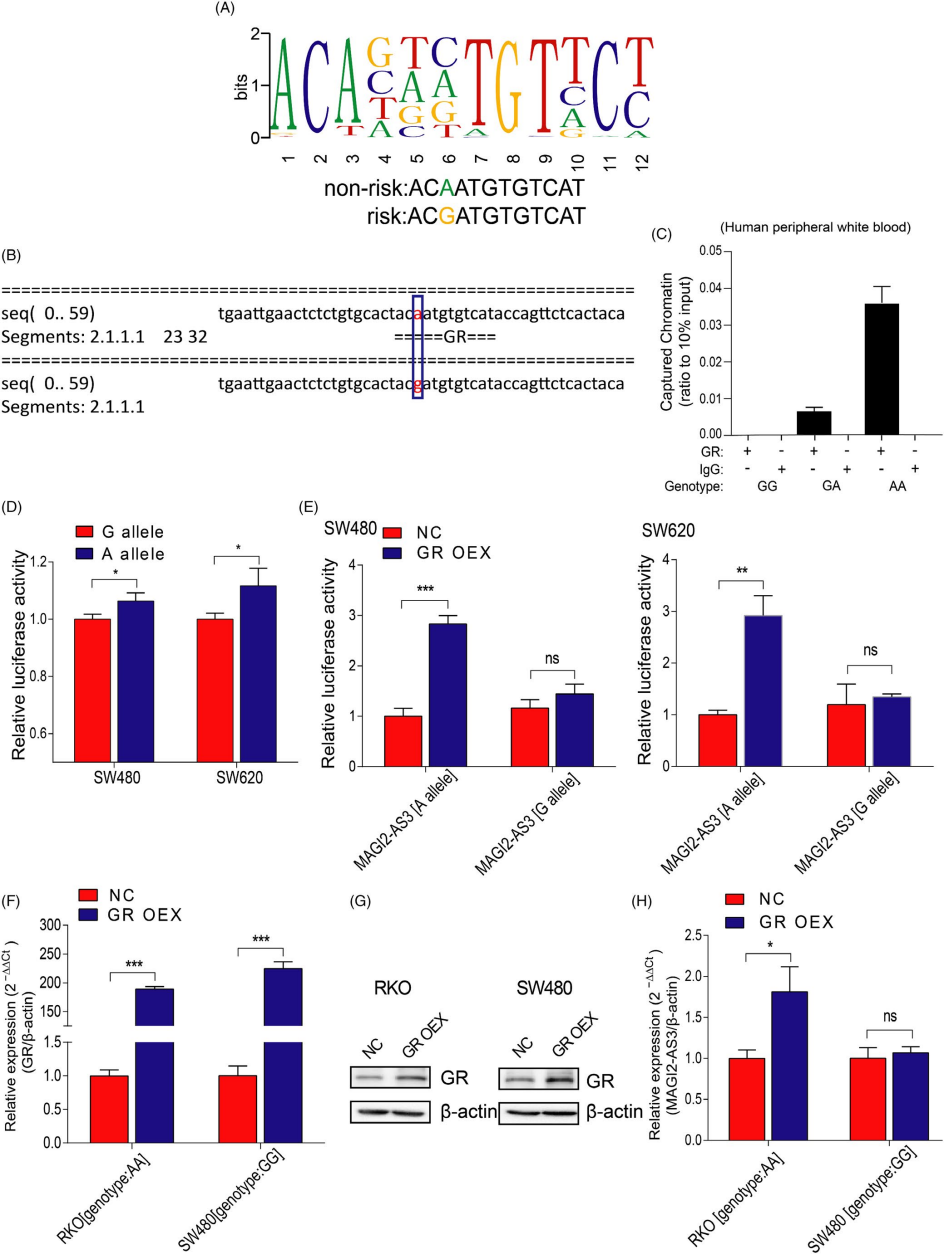
rs7783388 modulated GR binding to the MAGI2‐AS3 promoter region. A, Predicted preferential binding of GR to the non‐risk allele A of rs7783388 (ChIPBase 2.0). B, Prediction of the binding affinity of GR to the mutation region of rs7783388 with the bioinformatics tool (AliBaba2). C, The binding affinity between GR and the indicated MAGI2‐AS3 rs7783388 genotypes was determined by ChIP assay in CRC patient peripheral white blood. D, The effect of rs7783388 on MAGI2‐AS3 transcriptional activity was determined by luciferase reporter assay. E, The pGL3‐basic‐MAGI2‐AS3‐A‐allele or G‐allele construct as well as GR overexpression plasmid were co‐transfected into SW480 and SW620 cell lines. Relative luciferase activities in the indicated cells were determined. F, RT‐qPCR assay confirmed the efficiency of GR overexpression. G, GR protein level was significantly increased in RKO and SW480 cells transfected with GR overexpression plasmid compared to those transfected with NC plasmid cells. H, Relative expression of MAGI2‐AS3 was detected in RKO (genotype: AA) and SW480 cells (genotype: GG) transfected with GR overexpression plasmid. *P < .05, **P < .01, ***P < .001, ns: no statistical significance

Given that the SNP rs7783388 A > G mutation was located in MAGI2‐AS3 promoter region, we then analyzed MAGI2‐AS3 promoter using bioinformatics tools (ChIPBase 2.0 and AliBaba 2.1). We found that the rs7783388 A > G mutation might alter the binding affinity of GR to MAGI2‐AS3 promoter region (Figure 2A and B). To assess the efficacy binding affinity of GR to the MAGI2‐AS3 rs7783388 mutation region, we performed ChIP assay using CRC patients’ peripheral white blood. Results showed that GR preferentially binds to the MAGI2‐AS3 promoter with the rs7783388 AG/AA genotypes (Figure 2C). To further investigate the effect of rs7783388 on the interaction between GR and MAGI2‐AS3 promoter, we constructed the MAGI2‐AS3 promoter luciferase reporter plasmids (rs7783388 G allele or A allele) and transfected them into SW480 and SW620 cells alone or with GR overexpression plasmid. As Figure 2D shows, the cells transfected with rs7783388 A allele presented a higher luciferase activity than cells transfected with rs7783388 G allele. Moreover, GR overexpression significantly increased the luciferase activities of the cells transfected with rs7783388 A allele but not the cells transfected with rs7783388 G allele (Figure 2E). To explore the effect of GR on the MAGI2‐AS3 expression, we transfected RKO cells (genotype: AA) and SW480 cells (genotype: GG) with GR overexpression plasmid, respectively. The expression of GR was significantly upregulated by GR overexpression plasmid (Figure 2F and G). As Figure 2H shows, MAGI2‐AS3 expression was remarkably increased in RKO cells (genotype: AA) transfected with GR overexpression plasmid but not in SW480 cells (genotype: GG), indicating that GR may target MAGI2‐AS3 containing rs7783388 A allele but not MAGI2‐AS3 containing rs7783388 G allele. Altogether, we demonstrated that rs7783388 A > G polymorphism attenuated the transcriptional activity of MAGI2‐AS3 by downregulating the binding affinity of GR and then led to reduced transcription of MAGI2‐AS3 mRNA.

### 3.4 MAGI2‐AS3 overexpression attenuated CRC cell proliferation, invasion, and migration

**FIGURE 3:**
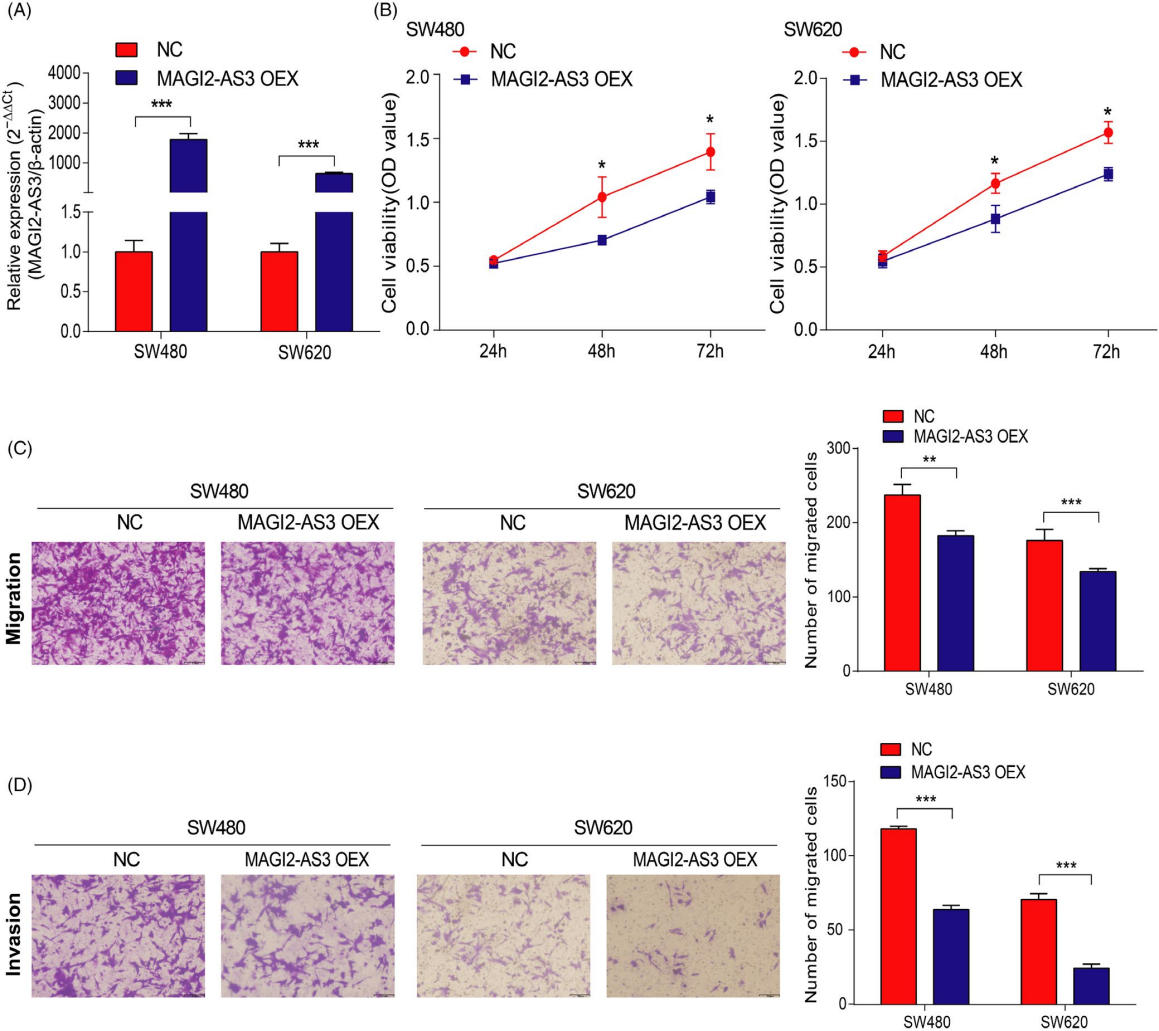
MAGI2‐AS3 overexpression inhibits SW480 and SW620 cell proliferation, migration, and invasion. A, RT‐qPCR confirmed efficiency of MAGI2‐AS3 overexpression. B‐D, Then, the effects of MAGI2‐AS3 overexpression cells on cell proliferation (B), migration (C), and invasion (D) were measured. *P < .05, **P < .01, ***P < .001

We next transfected MAGI2‐AS3 overexpression plasmid into SW480 and SW620 cells to examine the effect of MAGI2‐AS3 on CRC cells. The MAGI2‐AS3 expression level was effectively enhanced in SW480 and SW620 cells transfected with MAGI2‐AS3 overexpression plasmid (Figure 3A). MAGI2‐AS3 overexpression remarkably reduced cell proliferative capacity in CRC cells compared with NC groups, as measured by CCK‐8 assay (Figure 3B). Furthermore, transwell assay showed the significantly decreased invasion and migration abilities for CRC cells transfected with MAGI2‐AS3 overexpression plasmid (Figure 3C and D). These results supported that MAGI2‐AS3 could decrease proliferation, invasion, and migration capacities in CRC cells.

### 3.5 MAGI2‐AS3 overexpression promoted CRC cell apoptosis

**FIGURE 4:**
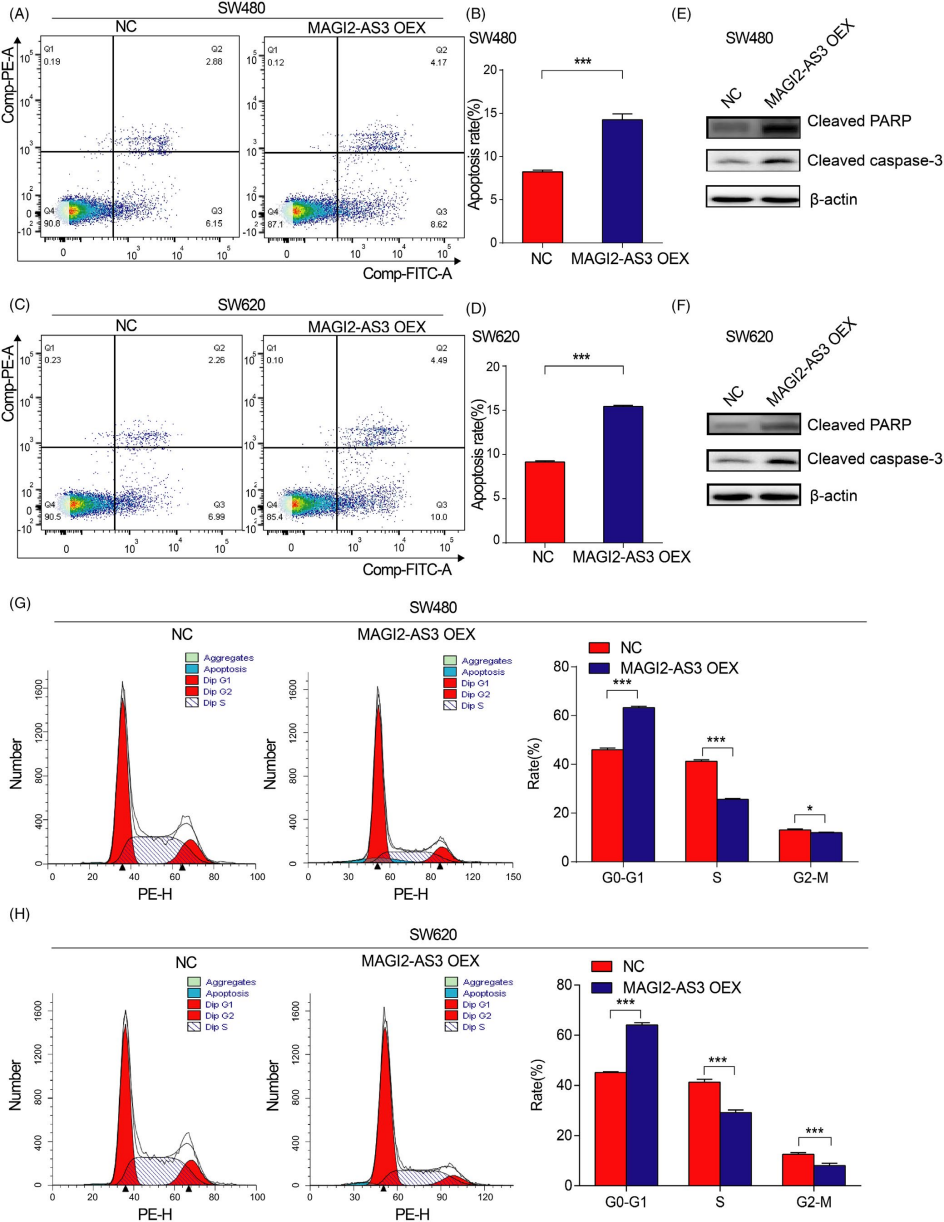
MAGI2‐AS3 overexpression induces the apoptosis of SW480 and SW620 cells. A‐D, Cell apoptosis analysis was conducted in SW480 and SW620 cells transfected with plasmid overexpression of MAGI2‐AS3 using flow cytometry. E‐F, Western blotting assays showed that cleaved PARP and cleaved CASP3 protein levels were significantly increased in SW480 and SW620 cells transfected with MAGI2‐AS3 overexpression plasmid compared to those transfected with NC plasmid cells. G‐H, Cell‐cycle analysis was performed in SW480 and SW620 cells transfected with plasmid overexpression of MAGI2‐AS3 using flow cytometry. *P < .05, ***P < .001

To explore whether the MAGI2‐AS3 contributes to CRC cell apoptosis, flow cytometric data showed that MAGI2‐AS3 overexpression significantly promoted apoptosis in both SW480 (Figure 4A and B) and SW620 (Figure 4C and D) cells. Western blot data displayed that the protein level of cell apoptosis–related genes (cleaved caspase‐3 and cleaved PARP) was all increased attributed to MAGI2‐AS3 overexpression (Figure 4E and F). In addition, compared with the NC groups, overexpression of MAGI2‐AS3 led to G0/G1 arrest in SW480 (Figure 4G) and SW620 (Figure 4H) cells. These results strongly revealed that MAGI2‐AS3 inhibited the tumorigenesis of CRC by promoting apoptosis and inhibiting the process of CRC cells from G0/G1 phase to S phase.

## 4 DISCUSSION

In the present study, we showed that rs7783388 GG genotype significantly increased the CRC risk when compared to AG/AA genotypes. Mechanically, the rs7783388 A > G mutation impacted the binding affinity of GR to the promoter region of MAGI2‐AS3, subsequently resulting in lower expression of MAGI2‐AS3, and ultimately promoting CRC development and progression.

In recent years, CRC with increasing morbidity and mortality rate has become one of the most common malignant tumors.^30^ It has been reported that several lncRNAs are aberrantly expressed in CRC and regarded as vital components of cancer risk.^32^, ^33^ For instance, Zhang et al^33^ identified the downregulation of lncRNA CPS1‐IT1 in CRC cells, and CPS1‐IT1 overexpression suppresses metastasis through inactivating HIF‐1α in CRC. lncRNA CASC9 expression was increased in primary colorectal cancer samples, and CASC9 initiates CRC development by regulating miR‐193a‐5p/ERBB2 axis.^34^ lncRNA RHPN1‐AS1 was upregulated, and further RHPN1‐AS1 could indirectly regulate OGT through sponging miR‐7‐5p and then promote CRC cell proliferation and invasion.^35^ Several studies have proved that MAGI2‐AS3 exerts an anti‐oncogenic effect in progression of human gliomas,^36^ hepatocellular carcinoma cell,^25^ and breast cancer.^24^ However, Ren et al^27^ reported that MAGI2‐AS3 drives colorectal cancer progression. In our study, we demonstrated that MAGI2‐AS3 played a protective role in CRC development. Although our finding differs from the literature, our research was in the most rigorous manner. First, qRT‐PCR data showed that compared with adjacent tissues, MAGI2‐AS3 was significantly lowly expressed in CRC tissues. In addition, the results derived from TCGA database were roughly consistent with qRT‐PCR data. Furthermore, MAGI2‐AS3 overexpression dramatically inhibited cell proliferation, invasion, and migration, caused G0/G1 cell‐cycle arrest, and simultaneously accelerated CRC cell apoptosis. These results highlighted that the MAGI2‐AS3 could function as a tumor‐inhibiting gene in the CRC development.

Recent studies have demonstrated that genetic mutations located in lncRNAs may have association with the susceptibility of CRC. For example, Shaker et al reported that the lncRNA HULC SNP rs7763881 A > C decreased the susceptibility of CRC by reducing the oncogenic HULC level.^37^ lncRNA RP11‐362K14.5 SNP rs1317082 T > C reduced the expression of CCSlnc362 by creating a bind site for miR‐4658, thus decreased the risk of CRC.^38^ A novel SNP rs10845671, locating in lncRNA RP11‐392P7.6 promoter region, was associated with the susceptibility of CRC.^39^ Our previous study has shown that MALAT1 rs664589 G allele upregulated MALAT1 expression by binding miR‐194‐5p and promoted CRC development.^40^ In the present study, subjects with MAGI2‐AS3 rs7783388 GG genotype have a higher risk of CRC compared with the AG/AA genotypes. qRT‐PCR and eQTL analysis results showed that MAGI2‐AS3 rs7783388 A > G variant leads to a decrease in MAGI2‐AS3 expression in CRC and adjacent tissues. These results further confirmed that SNPs in lncRNAs might contribute to CRC risk by affecting the expression and function of lncRNAs.

Glucocorticoid receptor is one of the best‐characterized metazoan transcriptional regulatory factors (TRFs).^41^ Recently, it has been reported that GR binds to TEAD4 promoter and promotes TEAD4 transcription during adipogenesis.^42^ Enguix‐Riego et al^43^ found that HSPB1 rs2868371 A > G promotes HSPB1 transcriptional level by promoting GR bind to the HSPB1 promoter region. Similarly, we obtained that the alteration from A to G at rs7783388 may modulate the binding affinity of GR to MAGI2‐AS3 promoter region. The expression of MAGI2‐AS3 was further enhanced when they were transfected with GR overexpression plasmids in CRC cells with AA genotype, not the GG genotype. We observed lower binding affinity of transcription factor GR at the risk allele G than the non‐risk allele A, thus downregulating MAGI2‐AS3 expression, and ultimately promoting CRC development. These observations indicate that SNPs in lncRNA promoter region are involved in CRC development by modulating the specific transcription factor–binding affinity to lncRNA promoter region.

In conclusion, we reported a risk SNP rs7783388 A > G could affect the transcription activity of MAGI2‐AS3, thereby modulating MAGI2‐AS3 level, further influencing the development of CRC.

## CONFLICT OF INTEREST

The authors declare no conflicts of interest.

## AUTHOR CONTRIBUTIONS

All authors contributed significantly to this work. Rui Chen, Xiaobo Li, and Xi Yang designed this study. Xi Yang and Shenshen Wu performed the experiments and were responsible for the data analysis. Xi Yang wrote the paper. Rui Chen and Xiaobo Li revised the manuscript. All authors read and approved the final manuscript.

## Supporting information

Fig S1

Tab S1

Figure Legend
