# Supplementary figures and images for: MAGI2‐AS3 rs7783388 polymorphism contributes to colorectal cancer risk through altering the binding affinity of the transcription factor GR to the MAGI2‐AS3 promoter
Source: J Clin Lab Anal. 2020 Jun 12;34(10):e23431. doi: 10.1002/jcla.23431 (PMC7595890; doi:10.1002/jcla.23431)

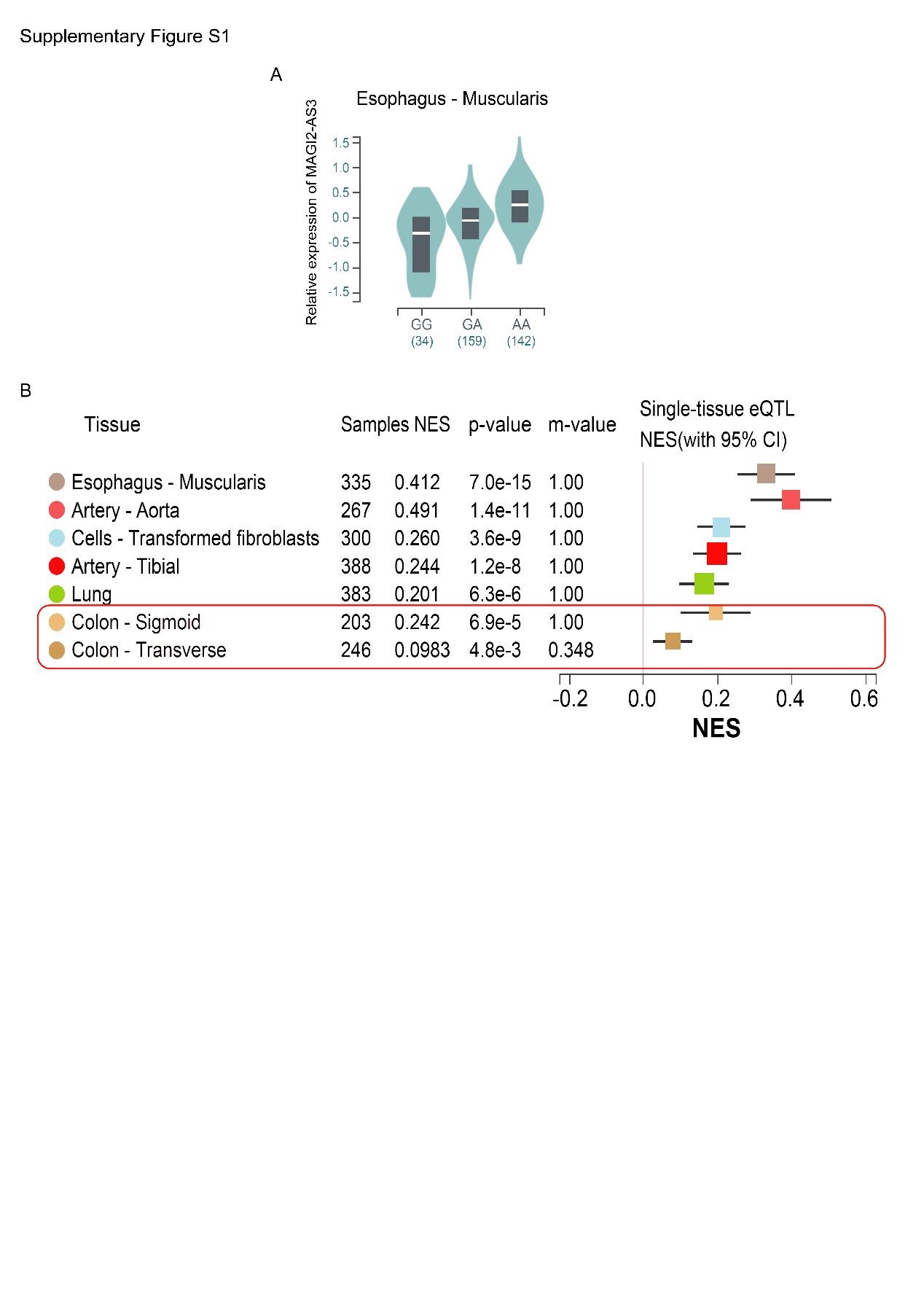

Supplement: Supplementary file 1 — Fig S1 [file JCLA-34-e23431-s001.docx]
