# Supplementary material for: MAGI2‐AS3 rs7783388 polymorphism contributes to colorectal cancer risk through altering the binding affinity of the transcription factor GR to the MAGI2‐AS3 promoter
Source: J Clin Lab Anal. 2020 Jun 12;34(10):e23431. doi: 10.1002/jcla.23431 (PMC7595890; doi:10.1002/jcla.23431)
Supplement: Supplementary file 2 — Tab S1 [file JCLA-34-e23431-s002.docx]

Supplementary Table S1 Frequency distribution of the selected variables in colorectal cancer cases and controls.

| Variables | Case (N = 1078） | | Control (N = 1175) | | P ^a^ |
| --- | --- | --- | --- | --- | --- |
|  | n | % | n | % |  |
| Age |  |  |  |  | 0.599 |
| ≤56 | 567 | 52.6 | 605 | 51.5 |  |
| >56 | 511 | 47.4 | 570 | 48.5 |  |
| Gender |  |  |  |  | 0.184 |
| Male | 659 | 61.1 | 686 | 58.4 |  |
| Female | 419 | 38.9 | 489 | 41.6 |  |
| Location |  |  |  |  |  |
| Colon | 475 | 44.1 |  |  |  |
| Rectum | 603 | 55.9 |  |  |  |
| Grade |  |  |  |  |  |
| Low | 356 | 33 |  |  |  |
| Intermediate/High | 722 | 67 |  |  |  |
| Depth of invasion | |  |  |  |  |
| T1 | 35 | 3.3 |  |  |  |
| T2 | 244 | 22.6 |  |  |  |
| T3 | 204 | 18.9 |  |  |  |
| T4 | 595 | 55.2 |  |  |  |
| Lymph node metastasis | |  |  |  |  |
| N0 | 621 | 57.6 |  |  |  |
| N1 | 457 | 42.4 |  |  |  |
| Distant metastasis | |  |  |  |  |
| M0 | 958 | 88.9 |  |  |  |
| M1 | 120 | 11.1 |  |  |  |
| TNM |  |  |  |  |  |
| I | 196 | 18.2 |  |  |  |
| II | 401 | 37.2 |  |  |  |
| III | 361 | 33.5 |  |  |  |
| IV | 120 | 11.1 |  |  |  |

^a^ Two-sided χ2 test for variables between cases and controls.

TNM: Tumor-node-metastasis.
