# Supplementary material for: MAGI2‐AS3 rs7783388 polymorphism contributes to colorectal cancer risk through altering the binding affinity of the transcription factor GR to the MAGI2‐AS3 promoter
Source: J Clin Lab Anal. 2020 Jun 12;34(10):e23431. doi: 10.1002/jcla.23431 (PMC7595890; doi:10.1002/jcla.23431)
Supplement: Supplementary file 3 — Figure Legend [file JCLA-34-e23431-s003.docx]

**Supplementary Figure Legends：**

**Supplementary Figure S1. The association between rs7783388 variant and MAGI2-AS3 expression.** (A) rs7783388 eQTL analysis of MAGI2-AS3 expression in esophageal muscularis tissues. (B) MAGI2-AS3 and rs7783388 eQTL in multiple tissues.
